# Progress of Veterinary Science

**Published:** 1885-04

**Authors:** 


### Progress of Veterinary Science.

INNERVATION OF THE BEAVER'S TAIL.—It is a remarkable and noteworthy fact, that while in the Rodentia generally—excepting *Dipus* and the *Leporidæ*—the lumbar enlargement is but feebly marked in the spinal cord, that this feature is exquisitely well developed in the beaver. The enlargement is most noticeable in the sacral nerve origins, and evidently connected with the intelligent use and muscular development of the trowel-like tail. E. C. S.

---

FOWL-DIPHTHERIA, a disease which has been studied in Germany by Dr. Klebs, has been prevalent this winter in Cairo. It is characterized by copious patches of membrane in the mouth and throat, swelling and ulceration of the neck, and inflammation of the intestines, with abcesses round the anus. Dr. Brugsch has been studying the disease microscopically, and by cultivation-experiments at the Khedivial Laboratory, which he has fitted up with the cultivation-apparatus which is used in Dr. Koch's laboratory. He has found in the membrane, and in the œsophagus, a bacillus resembling that found by Dr. Loeffler in the diphtheria of pigeons; but he has not yet found it in the other organs, nor has he succeeded in inoculating other animals with it.—*British Medical Journal.*

---

CASE OF GLANDERS.—M. Bucquoy has communicated to the Academie de Médecine a case of acute farcy in a coachman, which was treated in his wards at the end of last year. At the necropsy, there were no lesions observed of a special character. Inoculations made on a donkey with pus taken from an articulation of the patient provoked all the symptoms of glanders. Two incisions were made, one on the animal's nasal mucous membrane, the other on the thigh, and death ensued six days afterwards. These inoculations were made on the day that the patient died. The organic fluids collected during the life of the patient and used for artificial cultivations did not produce the typical bacilli described by MM. Capitan and Charrin. The necropsy of the donkey revealed all the characteristic lesions of glanders. Purulent mucus revealed from the animal's nostrils, and used for inoculating a guinea-pig, provoked acute farcy, and resulted in death after a few days. M. Leblanc instituted an inquiry in the stables where the patient had lived, and it was discovered that a horse suffering from glanders had been hidden there.

---

COW SURGERY.—Dr. J. M. H. Goodman, in *Gaillard's Medical Journal*, says: A valuable milk-cow received an injury, in crossing a railroad track, by an express train, midway between the knee and foot, making a severe lacerated wound, tearing away a large artery. I was unable to control the animal so as to apply a ligature, so made a compress and applied a roller bandage. Over this a thick coating of common pine-tar was applied, considerably below and above the wound, so that all air was successfully excluded, and over this another roller of cloth was applied. An examination of the wound was made in six days, and, to the astonishment of all who had witnessed the first dressing, the wound was almost entirely healed, and that without any suppuration. This wound was of such a serious character that t was proposed to kill the animal, but she was a very valuable cow for milk, and hence the effort, which resulted so favorably.

---

ACCIDENTS PRODUCED BY TINCTURE of ARNICA.—A veterinary surgeon,

M. Paul Cagny calls attention in the *Recueil de Medecine Veterinaire*, to the severe symptoms which are often produced in horses, particularly those with a delicate skin, by the free application of the tincture of arnica, and mentions the case of a foal which was kicked by another horse and in which the slight abrasion so produced was treated by the prolonged local application of bandages soaked in pure arnica. The part became swollen and inflamed, and soon had all the appearances due to the action of a powerful vesicant.—*Therapeutic Gazette.*

---

Prolapse of the Rectum in Swine cured by means of a Metallic Ring.—It is now thirteen years since I have adopted as a method of cure in prolapsed rectum the application of a metallic ring.

The first case which offered itself was that of a stout pig, about five years of age, in which the protruding rectum formed a tumor of the size of a goose egg. This was found to be covered all around with lacerations produced by friction with the sides of the sty. In order to lessen the size of the tumor free incisions were made and a considerable quantity of blood allowed to escape. Having smeared it well with olive oil I effected a reduction. The metallic ring was next introduced and retained by means of sutures implicating the entire thickness of the sphincter. I then ordered decoctions of linseed and marsh mallow and an oleaginous purge.

I visited the patient on the sixth day and found the cure established. The ring had fallen out and a thick circle of hypertrophied tissue, formed in consequence of the irritation produced by the ring, had taken its place.

I have treated many swine in this way and always with the best results.—*Mazzoli Luigi, V.S., in La Clinica Veterinaria.*

---

Use of Iodoform.—In a horse, ten years of age, castrated when a foal, the scrotal sac commenced to increase in size and become indurated, prominences appearing on its surface, which, in turn, became enlarged and ulcerated. I diagnosed sarcoma and extirpated the prominences, and applied powdered iodoform to the exposed surfaces. In two weeks the entire surface healed, and there has been no sign of a return of the disease since the operation was performed one year ago.

I extracted a hæmatoma from a dog's ear, and inserted into the wound a suppository of iodoform. Cure in ten days.—*G. Gioranoli, in La Clinica Veterinaria.*

---

Pulmonary Diseases in Wild Animals.—At a meeting of the Pathological Society of London, recently, Mr. J. Bland Sutton made an elaborate communication on this subject, in which he gave a general account of the Zoological distribution of certain forms of lung disease among wild animals dying in the Zoological Society's Gardens during the past three or four years. The opinion held by the medical profession and the world at large that wild animals in captivity die from pulmonary tuberculosis lacked foundation, and was certainly erroneous. The conclusions drawn were founded on the following cases. From October, 1881, to December 31st, 1884, the total number of deaths was 2779, made up of 583 mammals, 1408 birds, and 788 reptiles. Of the 583 mammals there were 303 quadrumana, including 7 anthropomorphous apes. Of these five mammals died from pulmonary tuberculosis—namely, a tree-porcupine, eyre, kinkajou, lagotis, and an agouti; only one case of general tuberculosis was seen, and that was in a coatimondi. It was remarkable that all of these tuberculosis animals came from South America and the tropical portions of North America, a region named by zoologists the "neo-tropical region." It differs from all the great zoological divisions of the earth's surface by the almost unequalled extent and luxuriance of its forests, its delightful climate, and the richness and variety of its animal life. It was also the home of the guinea-pig.

Pneumonic phthisis had been seen in twelve cases, of which five were monkeys and the rest carnivora. Even if these cases, by the utmost elasticity of the term "tubercle," be included, tuberculosis was a very infrequent cause of death in wild animals. The lungs in many of the cases of tubercle were examined by Dr. Heneage Gibbes for bacilli, and he found them present, not in thousands merely, but in millions. Dr. Gibbes has worked out some very important facts in connection with these micro-organisms, but this part of the research was left entirely in his hands, and the results will be published in a separate paper. Since mammals in confinement did not die from tuberculosis, it became necessary to give an account of their fatal diseases. It might be broadly stated that each group of animals had certain forms of chest affections common to the group. Thus primates, excluding man, suffered from bronchitis, atelectasis, and lobular pneumonia. Carnivora were exceedingly liable to double pleurisies, lobar pneumonia, and bronchitis; whilst ruminantia had the peculiar disease known as "Perlsucht" (the so-called bovine tuberculosis), bronchitis, and "worm" bronchitis. It will be seen that bronchitis, zoologically speaking, was widely diffused, and this was to be accounted for by the vicissitudes of the English climate, in contrast to the tropical climate, to which many of these animals were accustomed.

Although birds had been excluded from this report, so far as tuberculosis was concerned, yet there was one pathological condition peculiar to them which was of great interest. It was well known that in birds the bronchia were in communication with a series of membranous cavities known as air sacs. It happened, with especial frequency in water-fowl, that the lining membrane of these sacs inflamed, giving rise to exudation. This inflammatory matter coagulated, and often formed a covering half an inch in thickness, which formed an excellent nidus, wherein the mould penicillium might luxuriate and form a thin layer throughout the entire series of these air-chambers. Hunter, Owen, Müller, Robin, and others had noted the presence of mould in the air sacs of birds, but they all seemed to have overlooked the exudation. The interest of this condition lay in the fact that this mould did not confine itself to the air sacs, but even permeated the intercapillary air spaces of the bird's lung, which corresponded to the alveoli of the mammalian lung.

In view of these facts, was it a matter for wonder that, in the human lung, vegetable organisms, a fraction of the size of the spores of penicillium, requiring similar conditions for existence, occurred, such as were recognized under the name of bacilli. It was impossible to narrate all the details of the numerous cases of pulmonary affections which were to be observed. The "field of work" such an inquiry opened up to those who had the leisure and opportunity was immense.—*The Lancet.*

